# Bioactive Plant-Derived Compounds as Novel Perspectives in Oral Cancer Alternative Therapy

**DOI:** 10.3390/ph18081098

**Published:** 2025-07-24

**Authors:** Gabriela Mitea, Verginica Schröder, Irina Mihaela Iancu

**Affiliations:** 1 Department of Pharmacology, Faculty of Pharmacy, Ovidius University of Constanta, 900470 Constanta, Romania; gabriela.mitea@365.univ-ovidius.ro; 2 Department of Cellular and Molecular Biology, Faculty of Pharmacy, Ovidius University of Constanta, 900470 Constanta, Romania; 3 Department of Toxicology, Faculty of Pharmacy, Ovidius University of Constanta, 900470 Constanta, Romania; irina.iancu@365.univ-ovidius.ro

**Keywords:** bioactive natural compounds, cell signaling pathways, adjuvant therapy

## Abstract

**Background:** Oral squamous cell carcinoma (OSCC) is one of the most serious forms of cancer in the world. The opportunities to decrease the mortality rate would lie in the possibility of earlier identification of this pathology, and at the same time, the immediate approach of anticancer therapy. Furthermore, new treatment strategies for OSCC are needed to improve existing therapeutic options. Bioactive compounds found in medicinal plants could be used to support these strategies. It is already known that they have an increased potential for action and a safety profile; therefore, they could improve the therapeutic effect of classical chemotherapeutic agents in combination therapies. **Methodology**: This research was based on an extensive review of recently published studies in scientific databases (PubMed, Scopus, and Web of Science). The selection criteria were based on experimental protocols investigating molecular mechanisms, synergistic actions with conventional anticancer agents, and novel formulation possibilities (e.g., nanoemulsions and mucoadhesive films) for the targeted delivery of bioactive compounds in OSCC. Particular attention was given to in vitro, in vivo, translational, and clinical studies that have proven therapeutic relevance. **Results**: Recent discoveries regarding the effect of bioactive compounds in the treatment of oral cancer were analyzed, with a view to integrating them into oncological practice for increasing therapeutic efficacy and reducing the occurrence of adverse reactions and treatment resistance. **Conclusions**: Significant progress has been achieved in this review, allowing us to appreciate that the valorization of these bioactive compounds is emerging.

## 1. Introduction

Oral cancer represents one of the oncologic pathologies with increased incidence and mortality worldwide, being influenced by various factors such as chronic inflammation, smoking, alcohol consumption, low-antioxidant diet, human papilloma virus (HPV) infections, and genetic predisposition [1,2,3,4]. Over the years, the medical field has been characterized by significant advances made in the approach of conventional anticancer treatments (chemotherapy, radiotherapy, and surgery), and therefore, they are still the first treatment option for different stages of malignancy [5,6].

Since side effects and recurrence rates represent major concerns in clinical practice, numerous complementary and adjuvant therapeutic strategies have been explored and are on the increasing trend, including the use of natural compounds derived from medicinal plants [7,8,9,10], either in purified, synthesized, or nanoencapsulated forms, or as crude extracts [11,12,13]. These compounds, due to their involvement in the production of various mechanisms, such as regulation of signaling pathways involved in oncogenesis (PI3K/AKT, MAPK, NF-κB, etc.), induction of apoptosis and/or autophagy, and inhibition of proliferation by blocking the cell cycle, may exhibit significant antioxidant and anti-inflammatory effects [14,15,16].

There is a complex chemical profile of bioactive compounds that may offer promising insights into both therapeutics and prevention options for cancer, including oral cancer [17,18]. It is important to have a comprehensive knowledge of their chemical structure, to understand their biological mechanisms, and thus therapeutic targeting directions to focus on the development of synergies with conventional treatments that support and help in the validation of their contribution for clinical studies [19,20].

Thus, phenolic compounds and flavonoids are recognized for their antioxidant effects and modulation of molecular pathways related to inflammation and cell survival [18,21,22].

Other important classes are represented by that of stilbenes, with resveratrol as a representative example, known for its anticarcinogenic, antioxidant, and cardioprotective role [23,24], and quinones, and carotenoids that play a key role in protection against oxidative stress [25,26]. In parallel, numerous alkaloids possess potent cytotoxic and anti-proliferative effects and are frequently used as bases for antineoplastic drugs [21,27].

Terpenoids, present in essential oils, have gained significant attention due to their diverse pharmacological properties (antimicrobial, anti-inflammatory, and anticarcinogenic) and their alignment with the growing global interest in natural and sustainable products [28,29,30].

Understanding synergistic effects of plant extracts delves into this fascinating area of study, exploring how multiple bioactive compounds within medicinal plants interact to enhance or modulate their overall therapeutic efficacy [31,32,33].

To increase the potential therapeutic effect and reduce systemic toxicity, combinations of natural compounds with classical chemotherapeutic agents have been studied [34,35,36].

Also, research in the medical and pharmaceutical fields, due to continuous evolution, may support the development of therapies that can lead to increased efficacy through controlled delivery processes that can optimize the absorption of active principles from plants [37,38]. Polymeric drug delivery systems allow the introduction of an active substance with therapeutic properties into the body using devices or a newly designed formulation. Recent drug delivery systems emphasize biodegradable and bioreducible polymers with significant therapeutic advantages [39,40].

Thus, modern therapeutic approaches aim both to identify unique molecules with anticancer potential and to develop innovative formulations (nanoemulsions, mucoadhesive films, and liposomes) that improve bioavailability and thus lead to selective targeting of malignant cells [41,42,43]. Therefore, some compounds, such as quercetin, for example, have been incorporated into nanoparticles or orodispersible films, thus increasing local bioavailability and also increasing therapeutic efficacy in the oral cavity [44,45,46].

The purpose of this review is to present a detailed analysis of the different medicinal plants, natural extracts, and active principles that have been studied as treatment strategies for oral cancer. We identified relevant clinical and preclinical studies in the articles that reflected the mechanism by which these compounds act at the cellular and oral tumor level. Database research also highlighted the need to integrate complementary and innovative approaches in the treatment of these malignant diseases, with the aim of improving efficiency and safety in the patient’s treatment.

These studies suggested that plant-based therapies may offer a safer and potentially less invasive alternative to conventional treatments [16,47,48].

In this context, research in the field of plants and pharmaceutical treatments is becoming essential for the development of innovative therapeutic solutions to the current needs of patients with oral cancer [49,50,51,52].

## 2. Investigating the Therapeutic Potential of Bioactive Compounds in Oral Cancer

Compounds extracted from plants are promising directions in oncology research, aiming to develop innovative treatments for oral cancer [48,53,54,55]. Plant secondary metabolites are known for their essential roles in sustaining plant physiology, but their promising anticarcinogenic properties are now also being proven [56,57,58,59,60]. Through their ability to modulate cell signaling pathways and exert anti-inflammatory effects, bioactive compounds are emerging as potential therapeutics in oncology [58,61,62,63].

### 2.1. Categories, Compounds, and Biological Effects

#### 2.1.1. Phenolic Compounds

Phenolic compounds, recognized as bioactive secondary metabolites of medicinal plants, have attracted the attention of researchers due to their multiple biological effects, with an essential role in anti-inflammatory, antioxidant, and antimicrobial therapies [64,65,66] being characterized by the presence of polyphenols. Structurally, they can be described as compounds containing at least one phenol group, the phenol itself presenting a benzene ring substituted with a hydroxyl group, systematically known as hydroxybenzene [67,68].

Polyphenols are a subcategory of phenolic compounds, represented by polyhydroxylated bioactive compounds that encompass a wide variety of compounds with similar structures. They can be divided into several main subclasses such as phenolic acids, flavonoids, lignans, stilbenes, and tannins [69,70]. Polyphenols contribute to the intake of a multitude of nutritional micronutrients in our diet, with evidence of their beneficial role being supported by studies carried out to demonstrate their direct counteracting of oxidative stress and inflammation, two major key factors in oral carcinogenesis [71,72,73]. Phenols that contain carboxylic acid are called phenolic acids; among them are ferulic acid, caffeic acid, and transferulic acid [74,75].

#### 2.1.2. Flavonoids and Their Subclasses

Flavonoids are a class of naturally occurring secondary metabolites characterized by a polyphenolic structure and wide distribution in vegetables, fruits, as well as in various types of beverages [76,77,78]. Flavonoids are an important part of fields such as pharmacy and medicine, as well as cosmetics and nutraceuticals, due to their proven effectiveness in promoting and maintaining human health [79,80].

The beneficial effects of these compounds are attributed to their antioxidant, anti-inflammatory, and anticancer activities, along with their ability to influence the functioning of key cellular enzymes, recognized for their potential role in the management of oral cancer [76,81].

Flavonoids can be subdivided into different structural subclasses: anthocyanins, flavanones, catechins, flavonols, chalcones, flavanonols, isoflavones, and flavones that may further influence cancer cell behavior [82,83,84]. Flavones (apigenin and vitexin) are widely present in leaves, flowers, and fruits as glucosides [83,85,86].

Isoflavonoids are metabolites characteristic of leguminous plants and play essential roles in nodule induction and microbial signaling [87,88]. Isoflavones are grouped into three groups: genistein, daidzein, and glycytidine [89,90]. The molecular structure of isoflavones is like that of animal estrogens. In addition, isoflavones possess potent antioxidant activity, which may decrease the risk of cancer by inhibiting free radical-induced DNA damage [83,90].

#### 2.1.3. Stilbenes and Their Derivatives

Stilbenes and their derivatives, known as stilbenoids (pinosylvin and pterostilbene), represent a multidisciplinary research field that combines several important key branches of chemistry, biology, physics, and medicine [91,92,93]. They exhibit pronounced antimicrobial, estrogenic, and anticarcinogenic activities. Resveratrol, one of the best-known stilbene derivatives, is valued for its potent antioxidant activity, as well as for the moderate antimicrobial, fungistatic, and fungicidal properties of its derivatives [94,95,96]. These bioactive plant compounds demonstrate therapeutic value against oral cancer through their proven mechanisms of inducing apoptosis and inhibiting tumor growth [97,98,99].

#### 2.1.4. Flavonolignans

These compounds, flavonolignans, result from the combination of two phenylpropanoid units and present a distinctive structure that places them in the flavonoid class [100,101]. Silybum marianum flavonolignans, known as silymarin, are a complex mixture of structural constituents isolated from the fruits and seeds of the plant. They are recognized for their antihypertensive, hypolipidemic, antiatherosclerotic, and antidiabetic properties. Silymarin extracts have been used for centuries as traditional remedies for liver and gallbladder diseases [102,103,104,105]. Silymarin, also recognized for its anti-inflammatory and antioxidant effects, is a potential therapeutic candidate in reducing inflammation and oxidative stress associated with oral cancer [106,107,108,109]. Silymarin is a complex consisting of silibin, isosilibin, silidianin, and silicristin, widely used in the pharmaceutical industry and in dietary supplements [110,111,112].

#### 2.1.5. Quinone and Carotenoids

Quinone compounds, due to their unique chemical properties and their ability to participate in redox processes, have been recognized as promising and attractive alternatives in the field of anticancer drug development [113,114].

By targeting various cellular components in biochemical pathways, quinone compounds exhibit multiple mechanisms of action, inducing cytotoxicity and apoptosis in cancer cells, making them a promising option in the treatment of oral cancer [115,116,117].

Ubiquinol-10, the reduced form of coenzyme Q10 (ubiquinone-10), acts as a potent lipophilic antioxidant in various cell membranes and LDL and is also a well-known proton-electron transporter in the inner membrane of mitochondria [118,119,120].

Carotenoids are a group of secondary metabolites produced by the terpenoid biosynthetic pathway. These natural pigments, widely distributed in plants, fungi, algae, and bacteria, are responsible for their red, orange, and yellow coloration [121,122]. Carotenoids are recognized for their antioxidant role in a variety of diseases, including cancer [118,123]. Also, carotenoids produced by microorganisms contribute to stabilizing the cell membrane, thus strengthening its integrity [121,124].

#### 2.1.6. Alkaloids

Plants produce alkaloids, nitrogenous organic compounds characterized by their cyclic structure, the presence of an integrated nitrogen atom within the ring, and their alkaline properties [125,126].

Some alkaloids, such as amide alkaloids, can be found in free form in plants, being very weak alkaline, while others may have glycosidic forms with N-oxide groups. Some of the main classes of alkaloids found in plants are pyridine, pyrrolidine, quinoline, isoquinoline, quinazoline, steroids, and indole [127,128,129]. The bis-benzylisoquinoline alkaloids (e.g., tetrandrine) belong to the broader family of isoquinoline alkaloids, found predominantly in tropical and subtropical regions in plant families such as Menispermaceae, Berberidaceae, Lauraceae, and Ranunculaceae [130]. These alkaloids have shown significant research interest due to their anti-inflammatory, antitumor, and antiviral biological activities and increased potential in their pharmacological applications in conventional cancer therapies, including oral cancer [130,131,132,133,134,135,136].

#### 2.1.7. Essential Oils

Essential oils (EOs) are volatile liquids obtained by extraction from different plant parts. The bioactive compounds predominantly present are terpenes (monoterpenes, diterpenes) and terpenoids (monoterpenoids, diterpenoids, and sesquiterpenoids), which are recognized for diverse biological activities, including antimicrobial, anti-inflammatory, antioxidant, antiallergic, and anticarcinogenic effects [137,138,139]. Structurally, the sesquiterpene lactones (e.g., santamarine) are terpenes that share a common base structure of 15 carbon atoms being organized into several subclasses: eudesmanolides, guaianolides and pseudoguaianolides, germacranolides and xanthanolides [140,141].

#### 2.1.8. Phenylpropene

Simple phenylpropanoids serve as fundamental precursors in the biosynthesis of many complex natural compounds, such as flavonoids, lignans, and polyphenols. They are often associated with characteristic odors and may exhibit antimicrobial, anti-inflammatory, and anticancer effects [142,143]. These effects have also been associated with the phenylpropene t-anethole, one of these compounds, which provides the characteristic sweet flavor of anise seeds and leaves (*Pimpinella anisum*, family Apiaceae) [144,145,146]. These activities, through the reduction of inflammation and oxidative stress, may contribute to the modulation of the tumor micro-environment in oral cancer [147].

#### 2.1.9. Phthalides and Xanthones

Phthalides are found in some plant families and mushroom genera and are a small group of natural compounds that can be monomeric or dimeric in structure. They are known in Asia, Europe, and North America for their anti-inflammatory, antispasmodic, and sedative properties [148,149], also being very useful in reducing chronic inflammation associated with oral pathology [150,151].

Xanthones represent secondary metabolites with remarkable structural diversity, exhibiting a wide range of antitumor, antidiabetic, and antimicrobial pharmacological properties. These aromatic compounds, mainly found in higher plants such as Clusiaceae, Hypericaceae, and Gentianaceae [152,153,154], exert influence on cancer cell proliferation and apoptosis, thereby contributing to their therapeutic relevance in oral cancer treatment [155,156,157,158].

#### 2.1.10. Phytosterols

Phytosterols are known due to their chemical diversity, structural complexity, inherent biological activity, as well as their easy availability, accessibility, and lack of toxic effects. Besides their ability to inhibit intestinal absorption of cholesterol, which thus leads to a decrease in its level in plasma [159,160], phytosterols can be used as auxiliary agents in oral anticancer therapy [161,162,163]. Among the best-known forms of free plant sterols, found in significant amounts in vegetable oils and nuts, are campesterol, brassicasterol, β-sitosterol, and stigmasterol [164,165].

#### 2.1.11. Other Compounds

Cyclic peptides are polypeptide chains with a cyclic structure, possessing clinical importance for various biological activities, such as antibacterial or bactericidal (gramicidin, tirocidin, vancomycin) and immunosuppressive (cyclosporin A) [166,167,168].

Proteases are proteolytic enzymes that likely arose in the early stages of protein evolution, initially as simple destructive enzymes required for protein catabolism and amino acid generation in primitive organisms. Over time, these proteases led to complex functions influencing the regulation of gene expression, diverse physiological processes (ovulation, fertilization, hemostasis, blood clotting, stem cell mobilization), and regulation of cell homeostasis (inflammation, immunity, autophagy, necrosis, and apoptosis) [169,170,171].

The diverse biological activities and complex structure of these compounds (polyphenols, flavonoids, stilbenes, quinones, alkaloids, phytosterols) provide a rich source for future novel anticancer strategies in oral cancer pathology [18,172,173,174].

### 2.2. Phytotherapeutic Perspectives in Oral Cancer Pathology

The summary of the main characteristics of the included studies can be found in Table 1 (plant extracts), Table 2 (phytochemical compounds), and Table 3 (formulations/combinations of phytochemical compounds).

#### 2.2.1. Effectiveness of Plants and Natural Compounds Based on the Included Studies

In vitro tests have highlighted that most of the analyzed natural compounds, such as piperlongumine, α-mangostin, quercetin, and transferic acid, exert their action through similar molecular pathways, as described below [69,70,71,72].

A recent in vitro study investigating the anticarcinogenic effects of piperlongumine (PL) on the oral cell lines MC-3 and HSC-4 demonstrated that PL simultaneously activated apoptosis and autophagy, the two mechanisms involved in tumor cell death. Therefore, PL represents an effective therapeutic agent for the treatment of oral cancer. Further investigations to optimize dose and evaluate efficacy in combination with other autophagy inhibitors are needed to confirm previous findings [196].

In another study regarding α-mangosteen, found in *Garcinia mangostana* by using OSCC lines, the authors highlighted that this bioactive compound significantly inhibited the process of cell proliferation and participated in the induction of apoptosis. One of the valuable aspects in the oncology science observed in this study is that α-mangosteane presented a lower cytotoxicity compared to normal cells. Cell cycle phase arrest and inhibitory effects on mitochondrial apoptosis signaling pathways were also identified [206].

In addition to the previous research, a study was performed on quercetin, known for its antioxidant activity, which revealed that quercetin not only blocks proliferation but can also trigger cell death by endoplasmic reticulum stress (ER stress). Experiments conducted on the SAS cell line demonstrated that apoptosis is induced by the activation of proapoptotic proteins (CHOP/ATF) as well as by the release of cytochrome C from mitochondria [210].

In another study, the beneficial role of transferrin acid was highlighted by the activation of the caspase cascade, as a result of the reduction in the expression of antiapoptotic genes (Mcl-1) and the stimulation of the expression of proapoptotic genes (Bax). The conclusion of the study highlighted that this compound, in the early stages, can prevent tumor spreading through the essential mechanisms of blocking proliferation by mitochondrial mechano-mechanisms, and can be very valuable [202].

The flavonoid quercetin from propolis has been shown to potentiate tumor growth inhibitory activity. The beneficial effects of propolis in the treatment of oral cancer are the activation of immune effector cells such as cytotoxic T lymphocytes and macrophages, acceleration of cancer cell apoptosis, prevention of metastasis, anti-angiogenesis effect, mitosis-suppressing effect, immunomodulatory and antioxidant effect [230].

Another study has concluded that isoflavones from *Trifolium pratense* (red clover) could inhibit the proliferation of OSCC cells, with a relatively low toxicity profile [231].

In one research study on extracts from cranberry and grape seed, a selective apoptosis was induced, particularly in CAL27 and SCC25 cells, by activating the cell cycle inhibition process and increasing caspase activity by proanthocyanidins [182].

According to the authors, in a study that analyzed the performance of coenzyme Q10 (ubiquinone) and β-carotene in oral cancer patients, it was reported that T3 and T4 stages are directly proportional to antioxidant deficiencies, associated with both faster tumor progression and an altered metabolic status. Also, the same research suggested that antioxidant supplementation could partially improve the metabolic profile, with further studies needed to achieve a better understanding of the therapeutic benefits and optimize dosages [193].

Various bioactive compounds, such as silymarin, coenzyme Q10, and resveratrol, have begun to demonstrate clinical relevance. For example, silymarin (420 mg/day) reduced the severity and delayed the onset of radiotherapy-induced oral mucositis in both head and neck cancer patients [232], and systemic administration (140 mg three times per day) indicated a possible attenuation of chemotherapy-induced hepatotoxicity [233] although it remains a supplement without FDA approval [232]. Even though clinical studies on CoQ10, especially in OSCC, are limited, recent research has confirmed its safety and efficacy in preventing cardiotoxicity and decreasing cancer treatment-induced fatigue [234]. In a 2024 study, resveratrol induced feroptosis via the p53/SLC7A11 pathway in OSCC cell lines [191,229], and a March 2025 review supported its potent antiproliferative and antimetastatic actions in oral cancer models [235]; however, it does not hold drug approval [229].

Although the concrete results of the selected studies are presented in detail in this review, their limitations should also be mentioned. First, a major current limitation is the frequent use of non-standardized in vitro and in vivo models, which makes it difficult to directly compare the results. For example, nanoparticle penetration varies significantly between two-dimensional (2D) and three-dimensional (3D) models in OSCC [235,236]. Non-uniformity of dose, extract purity, and delivery systems in studies may lead to inconsistent results [237]. In addition, some information is conflicting, e.g., the dual role of antioxidants, as protectants but also as pro-oxidants, a role that may lead to cancer progression or inhibition depending on the context [238,239,240]. Due to the scarcity of high-quality clinical trials, future research should prioritize clinically relevant experiments.

#### 2.2.2. Synergism Between Natural Compounds and Conventional Medicines

Various studies show that co-treatment of thymoquinone with cisplatin enhances the selectivity and efficacy of the chemotherapeutic agent on oral malignant cells, also protecting some normal cells. The mechanism is based on the induction of apoptosis in tumor cells by altering the expression levels of pro/antiapoptosis-related genes (e.g., p53, Bcl-2, caspase) and even reducing the dose of cisplatin, which could help to decrease the associated systemic toxicity [225].

As a separate study, the synergy of anethole (a phenylpropene derived from essential oils) with the standard chemotherapeutic agent cisplatin was investigated in oral cancer cell lines. Studies have shown the apoptogenic potential of anethole in combination with cisplatin by inhibiting important molecular pathways involved in tumor progression (MAPK, NF-κB, and β-catenin). This combined effect may allow a lower dose of cisplatin to potentially allow a lower dose of cisplatin to have an efficient therapeutic response with reduced side effects and a more tolerable treatment overall [226].

#### 2.2.3. Advanced Topical Delivery Systems Used in This Specific Pathology

Many plant-derived compounds, such as flavonoids, stilbenes, and terpenoids, exhibit poor aqueous solubility, rapid metabolism, and low permeability, which limit their therapeutic potential in oral cancer treatment [241].

Recently, advances have been made showing that nanoparticles can significantly increase the bioavailability of natural products both in vitro and in vivo [40,242,243,244].

The stability, solubility, cellular uptake/internalization efficacy, specificity, tolerability, therapeutic index, and therapeutic efficacy of examples of natural compounds such as resveratrol and curcumin have been improved by integration into nanoemulsion-based drug delivery systems [245,246,247,248].

**Figure 1:**
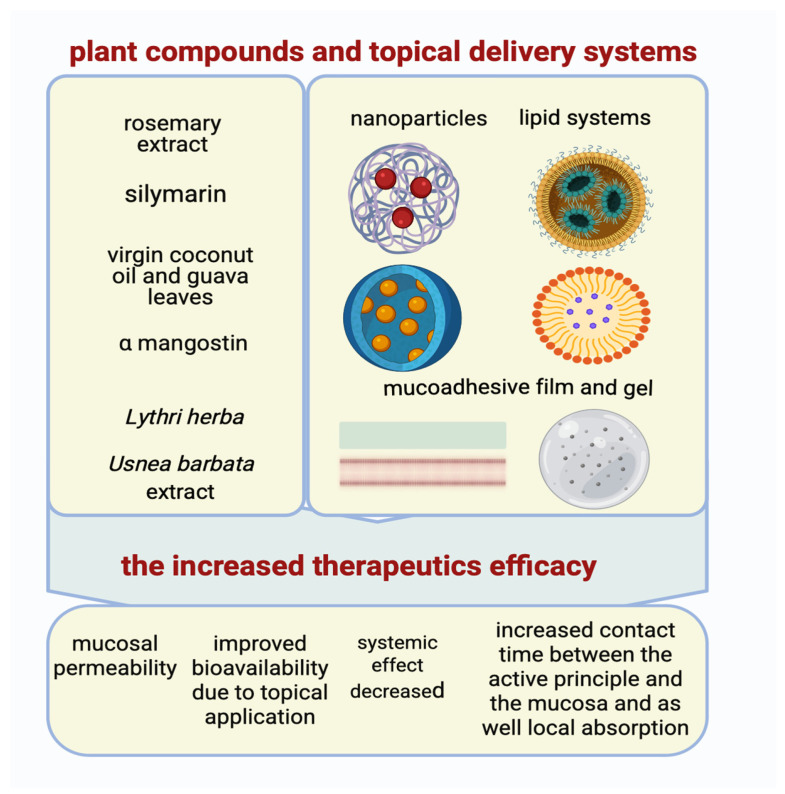
The advanced topical delivery systems and the enhanced anticancer activity of compounds (Created in BioRender. Schroder, V. (2025) https://BioRender.com/tz17glc, accessed on 29 June 2025).

A new modern area of high interest for researchers is the discovery of new formulation modalities (mucoadhesive gels, nano-encapsulated formulations, orodispersible films), which allow for direct topical application to damaged surfaces in order to increase absorption, therapeutic efficacy, and reduce systemic effects, as can be seen in Figure 1 [109,249,250].

Oral mucoadhesive films containing *Usnea barbata* (L.) F.H. Wigg extract, dispersed in canola oil, has established an increase in potential therapeutic effects in the complementary therapy of oral squamous cell carcinoma [249,250].

In our previously published articles, results showed a correlation between *Lythri herba* and chitosan concentrations and membranes’ swelling and stability, showing it could be a promising material for biomedical applications [251].

Our studies have also focused on incorporating cannabidiol oil as an active component in chitosan-based films, with the aim of identifying a new pharmaceutical application [252].

In one study, researchers encapsulated polydatin (e.g., PLGA), a stilbene derivative known for its chemopreventive actions, in nanoparticles to protect this bioactive compound from premature degradation. This procedure was used in experimental models of oral carcinoma, and the result showed a facile release of the compound in the oral area as well as an increased anticarcinogenic efficacy [221].

Other researchers have studied the increased stability of silymarin as a result of loading it into nanostructured lipid carriers (NLCs), which are subsequently incorporated into a mucoadhesive gel formed in situ. Optimization of the therapeutic effect and reduction of the occurrence of potential systemic adverse effects were sustained by increasing oral mucosal permeability and providing a targeted release [109].

In another research, the possibility of increasing the therapeutic efficacy of α-mangostin against oral cancer was studied. An orodispersible mucoadhesive film was formulated with direct application to the oral lesion, resulting in an increased contact time between the active principle and the mucosa as well as local absorption, in direct accordance with the occurrence of a reduced number of systemic adverse effects [222].

Another investigation focused on the incorporation of rosemary extract into chitosan nanoparticles, which showed enhanced diffusion to the oral tumor and limited diffusion to other tissues due to the increased stability of the bioactive substances [223].

In another example, a nanoemulsion, based on a combination of guava leaves and virgin coconut oils, was formulated and later integrated into an orodispersible film. The incidence of systemic effects was reduced, and the anticarcinogenic effect was potentiated as a result of improved bioavailability due to topical application [224].

These advanced delivery formulation technologies are imperative for solving bioavailability barriers and optimizing the therapeutic efficacy of natural compounds in oral cancer therapy [250].

## 3. Advanced Methods Used for Bioproducts Cellular Activity Assay

Natural products have been used for centuries in a diverse spectrum of healing systems due to their powerful immunomodulatory, anti-inflammatory, and antiviral properties [253]. In our previous studies, the antioxidant effect of aqueous extract obtained from *Prunus spinosa*’s dried fruits was tested by using it to reduce the level of the biomarker IL-6 in various forms of periodontitis [254].

**Figure 2:**
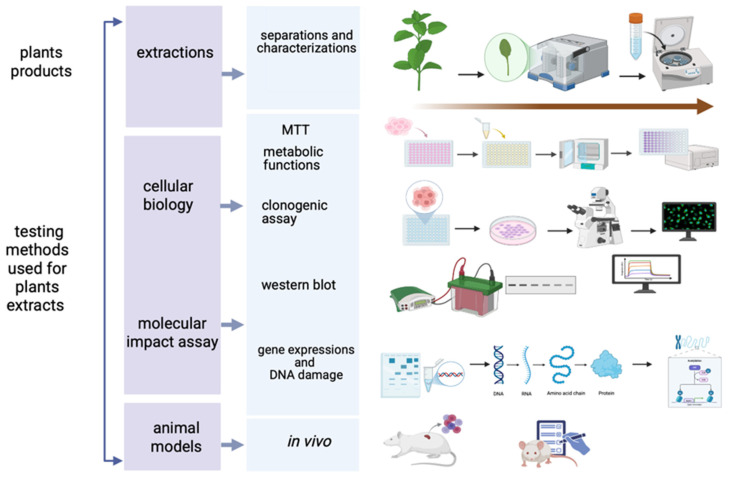
The testing methods used for the plant bioproducts cellular activity assay (Created in BioRender. Schroder, V. (2025) https://BioRender.com/4j8a7i6, accessed on 29 June 2025).

To evaluate the anticarcinogenic activity of phytochemical compounds on oral squamous cell carcinoma, multiple experimental methods have emerged over the last few decades (Figure 2) and have been applied in recent studies [187,188] (Figure 2).

Among the most versatile and popular assays used techniques are the MTT [255] assay for cellular metabolic activity, applied to oroselol [187], yohimbine [194], prenylflavones [195], piperlongumine (PL) [196], semilicoisoflavone B (SFB) [197], transferic acid [202], blumeatin [207], quercetin [209,210], sulforaphane [211], anethole [212], destruxin B [213], tetrandrine [136], β-sitosterol [217], burmanic acid (BURA) [220] and silymarin [106].

The clonogenic (or colony forming) assay, used for evaluating the radiation sensitivity of different cell lines [256], has been applied in studies on oroselol [187], prenylflavones [195] and quercetin [209,210], while Transwell assess cell migratory and invasive capacities [257] in relevant studies with oroselol [187], prenylflavones [195], quercetin [209,210], sulforaphane [211], blumeatin [207], kaempferol [208,216] and pinosylvin [216].

Western blotting has been one of the key methods in apoptosis and tumor progression for identifying specific proteins from a complex mixture of proteins extracted from cells by molecular weight [258]. This method was used for analysis PARP cleavage, LC3/BECN1 expression and caspases and has been used to investigate oroselol [187], resveratrol [189,190,191], prenylflavones [195], semilicoisoflavone B (SFB) [197], vitexin [199], blumeatin [207], quercetin [209,210], sulforaphane [211], anethole [212], destruxin B [213], cathepsin S [209,210], and tetrandrine [136] in the relevant articles in oral squamous cell lines. Corroborated with this method, similarly, α-mangostin [206], pinosylvin [216], CAPE [217,218], β-sitosterol [217], santamarin [219], burmannic acid (BURA) [220], and silymarin [106] demonstrated antitumor activity. In the case of lycopene, scrape-loading assays and electron microscopy demonstrated its superior ability to enhance gap-junction intercellular communication compared to β-carotene [215].

Flow cytometry has been used for analysis for the qualitative and quantitative assessment of cells in studies [259] for cell cycle and apoptosis involving yohimbine [194], prenylflavones [195], SFB [197], fisetin [203], blumeatin [207], quercetin [209,210], anethole [212], destruxin B [213], tetrandrine [136] and BURA [220].

For gene expression analysis, qRT-PCR was used for resveratrol [189,190,191], transferic acid [202], vitexin [199], and dehydroandrographolide (DA) [201]. Confocal microscopy allows for high-resolution imaging in thick tissues [260], and it was used to assess the effects of fisetin [203], quercetin [209,210], and sulforaphane [211].

Assessment of DNA damage was relevant in studies involving demethoxyymurrapanine (DEMU) [200], santamarin [219], and BURA [220], indicating a significant pro-apoptotic potential. In vivo studies have been tested for resveratrol [189,190,191], carnosic acid (CA) [204], DA [201], and silymarin [106], confirming antitumor activity in animal models.

## 4. Cell Signaling Mechanisms and the Trigger in Therapeutics Approaches

**Figure 3:**
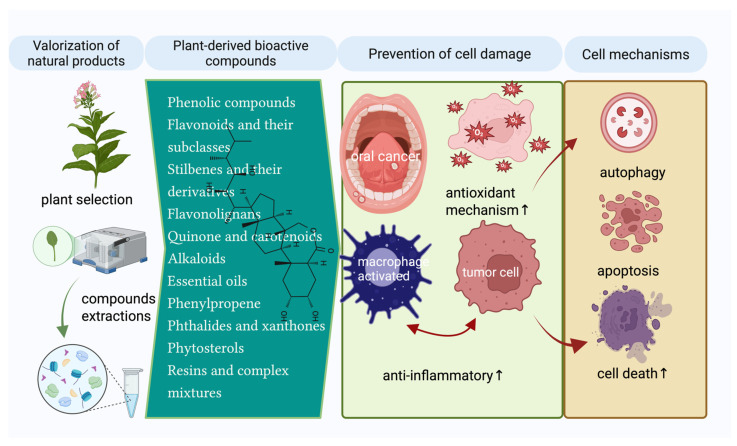
The main plant-derived bioactive compounds and cell modulating mechanisms are assigned (Created in BioRender. Schroder, V. (2025) https://BioRender.com/y5n3o3a, accessed on 29 June 2025).

The results of studies indicate a wide range of compounds whose general effects are aimed at preventing cell damage and, respectively, activating the anti-inflammatory defense mechanisms, triggering cellular mechanisms such as autophagy, apoptosis, or cell death necrosis (Figure 3).

This review article also addresses the cell signaling mechanisms by which plant extracts and bioactive compounds exert antiproliferative effects in oral cancer, especially in oral squamous cell carcinoma (OSCC), highlighted in multiple studies presented below. In addition, in the context of OSCC, compounds such as anthocyanins [188] and cannabinoids [198] have been analyzed in review articles for their role in molecular mechanisms and therapeutic benefits. Also, Z-Ligustilide has been evaluated in TW2.6 hypoxic oral cancer cells [205]. Ubiquinones and β-carotene have been explored in a clinical study, demonstrating therapeutic or preventive implications in OSCC [193].

Several relevant studies highlight the crucial role of signaling pathways in mediating the antiproliferative effects of plant extracts [179,180,181,182,186] and bioactive compounds such as demethoxymurrapanin, fisetin, carnosic acid, Z-ligustilides, etc. on OSCC carcinoma, highlighting its role in induction of mitochondrial apoptosis, activation of caspases-3, -8, -9, induction of BAX and/or inhibition of BCL2, DNA fragmentation and oxidative stress [200,203,204,205,207]. In addition, other compounds that follow the same principle of induction of programmed cell death by altering mitochondrial membrane potential, cytochrome c release, and activation of apoptotic cascades, such as α-mangostin, quercetin, β-sitosterol, burmannic acid, silymarin, etc., are mentioned in this review [106,206,209,210,213,217,218,219,220].

In a clinical study based on the use of silymarin, tumor growth inhibition, activation of DR5 receptor, and extrinsic apoptosis signaling pathway, a decrease in Bcl-2 levels followed by activation of caspases in succession was identified, which reinforced the pro-apoptotic characteristic of this natural compound. Another aspect highlighted the absence of significant hepatic and renal side effects [106].

In several current review articles, the authors present comprehensive results on the prevention and treatment of oral cancer using plant extracts, such as *Pinus densiflora* and *Trifolium pratens*, which act by inhibition of oncogenic transcription (e.g., STAT3), activation of MAPK/ERK, PARP, PUMA, and p53 pathways [176,181,231].

Also, other studies have observed that the methanolic extract of *Potentilla discolor* (MEPD) has a predominant effect by increasing pro-apoptotic PUMA expression and inhibiting STAT3 activation. In this regard, studies on STAT3 overexpression have highlighted the reduced therapeutic response for MEPD and the complementary apoptotic effect when combined with a STAT3 inhibitor such as crypto-tanshinone [176].

A major challenge in treating oral squamous cell carcinoma (OSCC) is drug resistance. Plant-derived bioactive compounds such as curcumin, resveratrol, and quercetin have been able to demonstrate their efficacy in this regard by inhibiting P-glycoprotein (P-gp), an essential efflux transporter encoded by MDR1. These compounds can improve drug sensitivity in resistant cancer cells by reducing drug efflux and increasing intracellular chemotherapeutic accumulation by down-regulating MDR1 and interfering with the ATP-binding domain of P-glycoprotein [261].

The taxonomy used for plants has been extracted from the reference https://www.ncbi.nlm.nih.gov/datasets/taxonomy/tree/ [175] (accessed on 24 April 2025).

Current researchers are focusing on the main signaling pathways based on stimulation of cellular and humoral immunity, including direct action on tumor cells that may be regulated by *Saraca indica* and *Momordica charantia* [183,184].

Extracts such as those of *Imperata cylindrica*, *Camellia sinensis*, and *Cardiospermum halicacabum* show antiproliferative effect on oral cancer cells, accompanied by apoptosis, cell cycle blockage, and DNA damage [177,178,185].

Underlining the potential of seaweed extracts as natural anticarcinogenic agents, another in vitro study with seaweed extracts showed that *Padina gymnospora* selectively targets oral tumor cells by upregulating pro-apoptotic signals and regulating the expression of many proteins involved in apoptotic pathways [180].

Recent systematic reviews have explored the potential of compounds such as oroselol, resveratrol, piperlongumine, semilicoisoflavone B, etc. in cell lines and some in vivo models, as therapeutics for oral cancer, highlighting their ability to induce apoptosis through various signaling pathways related to the induction of autophagy, apoptosis, and modulation of signaling pathways (PI3K/AKT, MAPK, NF-κB) [191,192,195,196,197].

This review focuses on highlighting several representative natural plant compounds—ubiquinones and β-carotene, transferic acid, propolis, etc.—that may lead to cancer cell death, for the regulation of pathways involved in antioxidant and anti-inflammatory effects, with action on oxidative stress and metabolism [193,194,200,202,230]. Extensive studies have further demonstrated the ability of some alkaloids, e.g., yohimbine, or flavonoids, such as prenylflavones from *Artocarpus altilis*, to induce programmed cell death in oral tumor cells that are resistant to conventional treatment [194,195].

The importance of natural products such as dehydroandrographolides, vitexin, resveratrol, and transferic acid in blocking numerous signaling pathways that favor carcinogenesis, such as inhibition of migration and invasion by suppressing metalloproteinase activity (MMP-2, MMP-9), and ERK1/2, NF-κB, AP-1 signaling pathways, and VEGF, u-PA expression is explored in reviewed articles [190,191,192,195,210].

Findings have indicated that cannabinoids can be used as compounds with immunomodulatory and CB1/CB2-specific receptor-regulating activity, inducing apoptosis and inhibiting proliferation of oral cancer cells [198].

Other mechanisms may support the prevention of tumor development and maintenance of tissue homeostasis through mechanisms underlying antiproliferation and increased intercellular communication, as can be demonstrated for lycopene [215].

While numerous plant-derived compounds show anticarcinogenic potential in oral squamous cell carcinoma (OSCC), their therapeutic use should be undertaken with caution due to reported toxicities. For example, silymarin, known for its antioxidant and anticancer effects in OSCC, may cause mild liver damage when administered in high doses or over extended periods [262]. Kavalactones in Kava extract, which have exhibited antiproliferative activity, are associated with hepatotoxic effects such as necrosis and drug-induced hepatitis [263]. Ginkgolic acids, known for their pro-apoptotic effects in cancer cells, carry significant risks including neurotoxicity, genotoxicity, and mutagenicity [264,265]. Moreover, resveratrol, long studied for its anti-inflammatory and anticarcinogenic properties in OSCC, can produce o-quinone metabolites that contribute to liver and kidney toxicity as well as skin disorders [266]. Such examples highlight the importance of extensive toxicological evaluations, standardization, and clinical validation to ensure the safety and efficacy of using plant-derived compounds in OSCC therapy.

These findings strongly support the argument for further studies, especially large-scale comparative studies and rigorous clinical trials, to test the natural compound’s role in oral carcinoma for their efficacy and safety. Integrating these compounds into contemporary treatment modalities, either as a single agent or in combination with established agents such as cisplatin, may provide less toxic and more precisely targeted adjuvant therapy options [37,225,226].

## 5. Materials and Methods

Comprehensive research literature published in prestigious international scientific journals, was performed through worldwide databases (PubMed, Scopus, SpringerLink, Google Scholar, Embase, Web of Science), using keywords such as: “oral squamous cell carcinoma”, “oral cancer”, “bioactive natural compounds”, “signaling pathways”, “adjuvant treatment”, “nanotechnology”. Selected studies were limited to publications within the last 5 years, i.e., between 2020 and 2025. This time limit was established to reflect the latest developments in the innovative herbal and pharmaceutical therapies for oral cancer. Priorities were given to in vivo and in vitro studies with clear evidence of the efficacy of these treatments.

## 6. Conclusions

In the literature, there is extensive evidence for many natural agents acting as supplements in the management of oral carcinoma. These mechanisms are predominantly cell cycle arrest, induction of apoptosis via multiple pathways, inhibition of migration/invasion, and local reduction of inflammation and oxidative stress under certain conditions. In addition, they are also very effective in combination with standard chemotherapeutic agents in enhancing their antitumor effect and decreasing their systemic toxicity.

However, most investigations are limited to preclinical studies (from in vitro to animal models), but there is a growing clinical interest in testing the efficacy and safety of these agents.

Future developments could involve nanoparticles and orodispersible forms, as well as controlled clinical trials to find out whether these phytocompounds can be commonly used as adjuvants to oral oncology treatments. Thus, natural agents could soon become real options for patients’ prognosis and quality of life.

These results highlight the need to further explore the optimization of new plant-based compounds for optimal delivery, bioavailability, and synergy with existing therapies.

In addition, one of its most important aspects was the possibility of synergism with conventional drugs that enhance the efficacy of a chemotherapeutic agent while minimizing side effects on normal cells.

## Figures and Tables

**Table 1:** Overview of plant-derived extracts and their importance in oral cancer treatment.

Scientific Name of the Plant	Family/ Class [175]	Type of Extract	Testing System	Cancer types/Methodology	The Importance of Results	References
*Potentilla discolor*	Rosaceae/Magnoliopsida	Methanolic *Potentilla discolor* extract (MEPD)	in vitro	-Extracellular Matrix (ECM) lines (MC3, YD15) -Trypan blue, Live/dead assay -Western blot: PARP, PUMA, p STAT3 -Immunofluorescence (PUMA, pSTAT3) -Gene transfection: STAT3 overexpression vector	-Highlights the STAT3- PUMA link in mucoepidermoid cancer apoptosis -Suggests MEPD as a complementary therapy -Shows the phytotherapeutic potential in the development of anticancer drugs.	[176]
*Imperata cylindrica*	Poaceae/Magnoliopsida	*Imperata cylindrica* leaf extract (ICL)	in vitro	-ICL methanolic extract (0–640 μg/mL) -SCC-9 vs. NIH/3T3 lines (fibroblasts) -MTT, clonogenic assay, flow cytometry (cell cycle) -DNA electrophoresis	-Demonstrates the potential of ICL against oral cancer -Support for the development of phytotherapeutic therapies with reduced toxicity -Basis for further studies	[177]
*Cardiospermum halicacabum*	Sapindaceae/Magnoliopsida	Plant extract	in vitro	-SCC25 cells (1.2 × 10^4^/well), treated with 25–125 μg/mL extract -MTT assay, with cyclophosphamide as positive control	-Suggests plant extract’s potential to fight oral cancer. -Higher doses, combinations with other substances, and in vivo studies need to be studied	[178]
*Scrophularia oxysepala*	Scrophulariaceae/Magnoliopsida	Essential oil	in vitro	-Tetrazolium staining method (viability) -Analysis of BAX, BCL2, SMAC, SURVIVIN gene expression by RT-PCR -Observation of cell morphological changes (apoptosis)	-*S. oxysepala* essential oil as a potential anticancer agent for oral cancer -Requires further investigation (in vivo testing) for clinical validation	[179]
*Padina gymnospora*	Dictyotaceae/Phaeophyceae	Seaweed extract	in vitro	-Treatment 15–20 µg/mL/24 h on KB- CHR-8-5 Cells -AO/EB staining for apoptosis morphology -Rh 123 and DCFH-DA for MMP and ROS -Differentially expressed proteins by 2D, MALDI- TOF/TOF -Bio-informatics analysis (STRING, PANTHER)	-Underlines the potential of *P. gymnospora* as a marine anticancer agent -Identification of the proteins involved helps in the development of future therapies based on natural products -Opens the way to in vivo validation research	[180]
*Pinus densiflora*	Pinaceae/Pinopsida	*Pinus densiflora* leaf essential oil (PLEO)	in vitro	-Steam distillation for extraction PLEO -YD-8 cells treated with 60 µg/mL, 8 h -Proliferation, cytotoxicity assays -ROS measurement, Western blot (caspase, Bcl-2) -Inhibitors (Vitamin E, z-VAD-fmk) to confirm the mechanism	-First study showing the anticancer potential of PLEO on YD-8 -Demonstrates a ROS-caspase pathway for apoptosis -Opens ways for the use of PLEO in the development of oral anticancer therapies	[181]
*Trifolium pratense*	Fabaceae/Magnoliopsida	Plant extract	in vitro	-Dried flower powder -MTT test on oral squamous cell carcinoma -Statistical analysis, linear regression for IC50 determination	-Confirms the potential of red clover as an alternative treatment for oral cancer -Further studies on detailed mechanisms are needed -Can be combined with other therapies for increased efficacy	[182]
*Cranberry* and *grape seed*	Ericaceae/Magnoliopsida and Vitaceae/Magnoliopsida	Plant extract	in vitro	-CAL27, SCC25 lines -Phenotypic tests: proliferation, adherence, morphology -Comparison of cranberry vs. grape seed effect -Evaluation of caspases, apoptosis	-Highlights the nutraceutical potential of fruit extracts in oral cancer prevention/treatment -Has dietary and complementary therapy implications -Requires identification of key components and clinical trials	[182]
*Saraca indica* bark	Fabaceae/Magnoliopsida	Methanolic extract	in vitro	-Wistar mice, dose groups 250 mg/kg and 500 mg/kg extract -Compared with levamisole and cyclophosphamide -Hematological parameters, DTH reaction, humoral antibodies, leukocytes, serum proteins -14 days	- Provides *Saraca indica* as a natural immunomodulatory agent -May be useful as an adjuvant in conditions of weakened immunity -Basis for further research on the mechanism and active substances	[183]
*Momordica charantia* (*Bitter melon*)	Cucurbitaceae/Magnoliopsida	Plant extract	in vitro and in vivo	-Laboratory studies (in vitro), animal models (in vivo) -Assessment of effects on proliferation, apoptosis, metabolism, and immune status -Limited epidemiologic analysis to support chemopreventive potential	-Bioactive substance with potential in the prevention/treatment of OSCC -May lead to the development of innovative chemoprevention strategies -Contributes to targeting future clinical research for confirmation of benefits	[184]
*Green tea*	Theaceae/Magnoliopsida	*Green tea* extract (GTE)	in vitro and clinical	-Review clinical studies/in vitro trials -Analyze effect of GTE on oral premalignant lesions, plus other cancers (breast, lung, colon, etc). -Monitor parameters: inflammation, apoptosis, proliferation	-GTE is an affordable, safe chemopreventive agent -Useful as a supplement in oral cancer prevention -Possible integration in screening programs/complementary treatments	[185]
*Pomegranate* extract	Lythraceae/Magnoliopsida	Standardized pomegranate extract (POMx)	in vitro	-Oral cancer cells (Ca9- 22, OC-2, HSC-3) vs. normal HGF-1 -Measurement of mitochondrial membrane potential, mitochondrial superoxide -Western blot, PCR (antioxidant genes, mitochondrial DNA) -Apoptosis (Annexin V, subG1)	-POMx as a selective anticancer agent for OSCC -Mechanism: mitochondrial oxidative stress -Requires preclinical/clinical studies to validate use in treatments	[186]

Legend: MEPD—Methanolic extract of *Potentilla discolor*; ICL—*Imperata cylindrica* leaf extract; PLEO—*Pinus densiflora* leaf essential oil; POMx—Standardized pomegranate extract; GTE—Green Tea extract; SCC-9, SCC25, CAL27—Human oral squamous cell carcinoma line; NIH/3T3—Mouse fibroblast cell line; KB-CHR-8–5—Drug-resistant human oral cancer cell line; YD-15, MC3—Human mucoepidermoid carcinoma cell line; YD-8—Human oral squamous cancer cell line; HGF-1—Human gingival fibroblast cell line; MTT assay—colorimetric assay for measuring cellular growth; RT-PCR—reverse transcription—polymerase chain reaction; 2D and MALDI-TOF/TOF—two dimensional and matrix-assisted laser desorption/ionization time of flight/time of flight; AO/EB—Acridine orange/Ethidium bromide; Rh123—Rhodamine 123; DCFH-DA—2′,7′-Dichlorofluorescin diacetate; Annexin V/SubG1—protein commonly used to detect early apoptosis in flow cytometry/cell fragments, often as a result of apoptosis; PARP—Poly (ADP-ribose) polymerase; PUMA—p 53 upregulated modulator of apoptosis; STAT3—Signal transducer and activator of transcription 3; BAX-BCL2—pro-apoptotic protein; SMAC—second mitochondria-derived activator of caspases; SURVIVIN—apoptosis inhibitor; IC50—half-maximal inhibitory concentration; DTH—delayed-type hypersensitivity; ROS—reactive oxygen species; MitoMP—mitochondrial membrane potential; z-VAD-fmk—cell-permeant pan-caspase inhibitor; ECM—epithelial carcinoma, mucoepidermoid; DNA—Deoxyribonucleic acid; OSCC—Oral Squamous Cell Carcinoma; MMP—mitochondrial membrane potential; Ca9-22, OC -2, HSC -3—Oral cancer cells; PCR—polymerase chain reaction.

**Table 2:** Overview of phytochemical compounds and their importance in oral cancer treatment.

Phytochemical Compounds	Class of Compounds	Methodology/Cancer Types	The Importance of Results	References
Oroselol	Phenolic compound	-Colorimetric test that measures activity of mitochondria (MTT assay), (viability, doses 15–120 µM, 48–72 h) -Clonogenic test (colony formation) -Transwell for migration/invasion -Western blot (LC3, p62, PI3K/AKT phosphorylation) -ANOVA *p* < 0.05	-Demonstrates the potential of oroselol as an anticancer agent (oral carcinoma) -Highlights the role of autophagy and the PI3K/AKT pathway in oral carcinoma -Provides a mechanistic basis for novel chemoprevention strategies	[187]
Anthocyanins	Flavonoid	-in vitro, animal studies, OSCC data.	-Anthocyanins are affordable, safe, and potentially chemopreventive -Supportive in decreasing the risk of OSCC, possibly adjunctive therapy -Extensive clinical studies are needed to confirm bioavailability and efficacy	[188]
Resveratrol	Stilbene	1. -In vitro (Ca9-22) and in vivo (nude mice) models siRNA for E-FABP, SREBP1 -qRT-PCR, RNAscopes, luciferase -Autophagy inhibitors, viability assays 2. -Mucosal fibroblasts exposed to arecolin ± resveratrol -Measurement of α-SMA, collagen I, ZEB1, miR-200a -Cell migration assays, contractility -Statistical analysis *p* < 0.05 3. -YD-10B cells, treated with resveratrol at various concentrations. -MTT (viability), immunoblot (TWIST, SLUG, E-cadherin); -Invasion assay (Boyden, Matrigel) -Statistical analysis *p* < 0.01 4. -Systematic review (PRISMA guidelines) + meta-analysis -Five studies were included in the statistical analysis -Assessment of neoplastic parameters: proliferation, apoptosis, overall effect size (ES) -Subgroups analyzed (cell type, exposure)	1. -Points to SREBP1 as a new therapeutic target—Resveratrol as a selective inhibitor of tumor lipid metabolism. Possible adjuvant treatment for oral cancer 2. -Possible adjuvant therapy for OSF (precancerous)Prevents progression to oral carcinoma -Clear molecular mechanism: miR-200a 3. -Demonstrates the potential of resveratrol to block EMT in oral cancer -Can be used as a complementary natural therapeutic agent, possibly with existing chemotherapeutic 4. -May be an effective complementary agent in oral cancer prevention/treatment -Needs larger clinical trials for confirmation. -Supports integration of resveratrol into multifactorial therapeutic approaches	[189,190,191,192]
Ubiquinones and β-carotene	Quinone/Carotenoide	-194 oral cancer patients, separated according to TNM stages -Measurement of vit. Antioxidants (CoQ10, β-carotene), antioxidant enzymes, Metabolic parameters (lipids, glycemia), inflammation markers (CRP, IL-6) -Statistical analysis (correlations *p* < 0.05)	-Suggests antioxidant supplementation in patients with OSCC -Highlights the link between metabolic disorders and cancer progression -Supports personalized nutritional interventions	[193]
Yohimbine	Alkaloid	-MTT assay (IC50~44 µM) -Morphological observations (shrinkage, blebbing) -Measurement of ROS, mitochondrial potential (flow cytometry) -Concentrations 40–50 µM yohimbine	-Shows potential of yohimbine against oral-resistant cancer -Example of drug repurposing -Requires in vivo studies for validation	[194]
Prenylflavones	Flavonoid	-Compound isolation/purification (chromatography, NMR, ESI-HRMS) -MTT tests on OSCC lines (SAS, T.Tn) + normal HaCaT cells -Molecular docking + dynamic simulations -Clonogenic assay -Flow cytometry (cycle block, apoptosis) -Wound healing assay (migration	-Demonstrates the potential of prenylflavones studied as anticancer agents with high selectivity for OSCC. -Anti-proliferative, anti-metastatic, and pro-apoptotic efficacy -Leads to the development of targeted prenylflavone-based therapies from *Artocarpus altilis*	[195]
Piperlongumine (PL)	Alkaloid amide	-In vitro, MC-3, HSC-4 lines -Viability assays, Western blot (p38, JNK, ERK) -Autophagy blocked with hydroxychloroquine (HCQ) -Assessment of apoptosis, cytotoxicity	-PL has significant anticancer potential in oral cancer -Causes apoptosis and cytoprotective autophagy -Combination with autophagy inhibitors may improve therapeutic efficacy	[196]
Semilicoisoflavone B (SFB)	Isoflavonoid	-MTT assay, flow cytometry (G2/M phase, Annexin V) -Western blot apoptosis proteins (Bax, Bcl-2, caspases) -NAC to block ROS -MAPK, Ras, Raf, MEK (phosphorylation) assessment	-SFB is a robust anticancer agent for OSCC -Complex mechanism: ROS, cycle blockade, MAPK Signaling -May be used clinically for OSCC management, requires further studies	[197]
Cannabinoids	Terpenophenolic compounds	-Review of epidemiological data + in vitro studies on oral cancer cells -Analysis of cannabinoid regulated signaling pathways	-Can be used as adjuvant therapy with anticancer benefits + reduced side effects -Studies still needed to clarify risks vs. protection -Could improve the quality of life for oral cancer patients	[198]
Vitexin	Flavone	-p53 inhibitor (PFT-α) for p53- dependent confirmation -PCR/Western blot p53, p21, Bax -MAPK inhibitor (PD98059) -MMP-2, PAI-1 analysis -viability, metastasis test	-Highlights the p53-dependent pathway in vitexin action -Demonstrates anti-cancer and anti-metastatic potential of vitexin in OSCC -Vitexin could be a promising therapeutic agent, requiring further studies	[199]
Demethoxymurrapanine (DEMU)	Alkaloid	-Ca9-22 lines, CAL 27 (oral cancer), vs. normal cells (S-G) -Treatment 0–4 μg/mL DEMU, 48 h -Measurement of ROS, superoxide, glutathione -Evaluation of subG1, Annexin V, caspases 3/8/9 -DNA damage (γH2AX, 8-OH-dG) -NAC pt. testing the role of oxidative stress	-DEMU acts preferentially on OSCC cells, having anticancer selectivity -Potential therapeutic agent with minimal adverse effects -Further studies needed for clinical applicability	[200]
Dehydroandrographolide (DA)	Diterpenoid lactone	-SCC9 lines -Wound closure, Boyden chamber (migration/invasion) -Gelatin zymography + Western blot MMP-2 -PCR MMP-2, TIMP-2, NF-κB, AP-1, SP-1 -In vivo xenograft model for metastases	-DA has important anti-metastatic potential in oral cancer -May be used to prevent tumor spread -Offers an alternative/complement to classical therapies, requires clinical trials	[201]
Transferulic acid	Phenolic acid	-MTT assay (20–120 μg/mL, 24–48 h) -DAPI staining (nuclear morphology) -qRT-PCR (Bax, Mcl-1) -FACS/PI for cell death	-Demonstrates major anticancer potential by activating apoptosis in OSCC -Can be used alone or in combination with other treatments -In vivo studies needed for other cancers	[202]
Fisetin	Flavonoid	-Flow cytometry (ROS, Ca^2+^, caspases) -DAPI staining (chromatin condensation) -Comet assay (DNA lesions) -Western blot apoptotic proteins -Confocal microscopy (cytochrome c, AIF, ENDO G)	-Fisetin is a potential anticancer agent in OSCC -Multiple mechanisms => receptivity to combination therapies -Basis for the development of novel treatments	[203]
Carnosic acid (CA)	Diterpenoid	-In vitro: CAL27, SCC9 (proliferation, migrationROS, Ca^2+^, MMP assays) -In vivo: BALB/c nude mice, xenotransplantation (CAL27/SCC9) -Western blot apoptotic proteins, tumor histology	-CA has remarkable therapeutic potential as it is safe and effective -Can be combined with other treatments -Helps in understanding mitochondrial mechanisms	[204]
Z-Ligustilide	Phthalide compound	-TW2.6 cells (hypoxic oral cancer) -Treatments with ligustilid + c-Myc/IRE1α inhibitors -Morphology, viability, migration analysis -γ- H2AX for DNA Damage -Combinatorial studies with radiation	-Potential therapeutic agent in oral cancer, including under hypoxia -May act as a sensitizer to radiotherapy -Requires future studies for combination therapies (ligustilide + radiation)	[205]
α-Mangostin	Xanthone	-Treatment with α-mangostin on OSCC cells -Apoptosis assessment (nuclear fragmentation, annexin V/PI) -Mitochondrial membrane potential analysis -Cellular cycle study (CDK/cyclin)	-α-Mangostin could be an effective therapeutic agent with low toxicity -Acts via mitochondrial mechanism and G1 cycle arrest -Proposed as a complementary therapy in oral cancer	[206]
Blumeatin	Flavonoid	-MTT assay (0–200 μM) on SCC-4 and hTRET-OME -Transwell (migration, invasion), wound healing-TEM for autophagy -Flow cytometry (ROS, MMP) -Western blot (LC3B, p62, Beclin 1)	-Blumeatin is a potent anticancer agent against OSCC -Also shows anti-metastatic effect (inhibits migration/invasion) -Higher selectivity against normal cells	[207]
Kaempferol	Flavonoid	-Migration/invasion assays (SCC4 cells) -Analysis of mRNA/proteins MMP-2, TIMP-2 -MMP-2 transcription study, c-Jun activity -ERK1/2 phosphorylation measurement	-Kaempferol has metastasis prevention/treatment potential for OSCC -Molecular approach: blocking c-Jun, ERK1/2 -Possible to integrate into complementary clinical applications	[208]
Quercetin	Flavonoid	1. -Flow cytometry (Annexin V/PI, ROS, Ca^2+^) -Western blot apoptotic proteins (Bcl-2, Bcl-XL, Fas, casp-8 etc.) -Confocal microscopy for cytochrome c -Treatment interval 6–48 h 2. -KON cancer lines vs. MRC-5 normal fibroblasts -MTT test(cytotoxicity, 1.5625–200 µg/mL), 24–96 h -Morphological analysis (nuclear condensation) -Colony formation test -Migration/invasion test (Transwell) -ANOVA + Tukey post-test	1. -Quercetin can be a promising therapeutic agent for oral cancer -Induces apoptosis via multiple pathways (ER + mitochondria) -Opens the way for future formulations/therapies in OSCC management 2. -Quercetin may be an adjuvant in standard OSCC therapy -Showing selectivity, lower adverse effects -Requires thorough mechanistic studies and in vivo confirmation	[209,210]
Sulforaphane	Isothiocyanate	-SCC-9, SCC-14 cells, treated with 0–10 μM sulforaphane (24–48 h) -MTT (viability), wound healing, Boyden (migration/invasion) -Western blot (cathepsin S, LC3, phosphorylated ERK1/2) -Confocal microscopy (GFP-LC3 spots)	-Sulforaphane may be a potential oral anticancer therapeutic agent -Regulation of cathepsin S and autophagy is a new target -Substance derived from cruciferous vegetables, supporting the idea of dietary role in oral cancer prevention/treatment	[211]
Anethole	Phenylpropene	-Malignant gingival cells (Ca9-22) -MTT, LDH assays for viability/proliferation -Apoptosis, autophagy, and ROS measured by flow cytometry -Western blot (p53, p21, caspase, NF-κB, etc.) -migration/healing assay	-Potential selective anticancer therapeutic agent, minimal impact on normal cells -May limit metastasis by blocking EMT -Useful as an adjuvant/complementary in existing cancer therapies	[212]
Destruxin B	Cyclic peptide	-Inhibits viability of GNM lines, TSCCa (oral cancer) vs. normal gingival fibroblasts -MTT test (viability) at 24–72 h -Annexin V/PI, caspase-3 immunofluorescence -Western blot (Bax, Bcl-2, caspase-3)	-Destruxin B proves selective for oral cancer cells -Promising safety profile -Possible complementary agent in oral cancer therapies (e.g., metastatic forms)	[213]
Cathepsin S (CTSS)	Protease enzyme	-OSCC lines, MP treatment -Cell viability, cell cycle (G2/M) -Western blot (caspase, PARP) -Autophagy study (LC3, beclin-1) -CTSS + p38/JNK role analysis	-Demonstrates a complex mechanism (apoptosis + autophagy -CTSS becomes a potential target for combination with MP -Offering new therapeutic strategies in OSCC, increasing tumor sensitivity	[214]
Tetrandrine	Bisbenzylisoquinoline alkaloid	-MTT assay (viability) -DAPI, Annexin V/PI (apoptosis -Western blot (caspase, PARP, LC3, Atg-5) -Autophagy inhibitor studies (bafilomycin A1, 3-MA, chloroquine, NAC)	-A dual mechanism (apoptosis + autophagy) for tetrandrine is evidenced -Potential multifunctional anticancer agent, synergy with autophagy inhibitors of interest -Opens the way to further clinical applications in OSCC	[136]
Lycopene	Carotenoid	-Lycopene/beta-carotene- treated KB-1 cells -Measurement of proliferation, connexin 43 expression (PCR, Western blot) -Gap-junction assay (scrape-loading, electron microscopy) -Carotenoid uptake analysis	-Lycopene has greater anticarcinogenic potential than beta-carotene in OSCC -Enhances intercellular communication (connexin 43), decreasing carcinogenesis -Supports the role of nutrition (tomato, carotenoids) in preventing oral cancers	[215]
Pinosylvin	Stilbene	-SAS, SCC-9, HSC-3 lines treated with 0–80 μM pinosylvin -Western blot (MMP-2, TIMP-2, ERK1/2) -Gelatin zymography for MMP-2 activity -Wound healing + Transwell (migration/invasion)	-Pinosylvin has anticancer potential, preventing OSCC metastasis -Contributes to the development of therapies targeting MMP-2 and ERK1/2 -Could be an adjuvant agent in the prevention of metastasis	[216]
Caffeic acid phenethyl ester (CAPE)	Phenolic acid	-Cell viability: trypan blue, live/dead assay -Soft agar assay for neoplastic transformation -Western blot (caspase-3, PARP, Bax, Puma) -DAPI staining for nuclear fragmentation	-CAPE as a promising agent in oral cancer treatment -Clarifies the role of Bax/Puma in apoptosis -Can be used in complementary therapies with reduced side effects	[217,218]
β-Sitosterol	Phytosterol	-Cytotoxicity, MTT assay (KB cells) -Flow cytometry for apoptosis confirmation -mRNA analysis (caspase-3, caspase-9, BAX, BCL-2) -Bioinformatics (STITCH, docking) for compound–protein interactions	-Natural oral cancer therapeutic agent, potentially safer -May reduce side effects of conventional therapies	[217]
Santamarine	Sesquiterpene lactone	-OC-2, HSC-3 cells (cancerous) vs. S-G (normal) -Measurement of ROS, mitochondrial superoxide, and GSH -Apoptosis (cytometry, Western blot, caspase) -DNA analysis (γH2AX, 8-hydroxy-2-deoxyguanosine (8-OH-dG)) -NAC effect (antioxidant)	-Substance with selective oral anticancer potential -Oxidative stress-type mechanism -> cell death -Basis for preclinical studies, capitalizing on the natural source, *Michelia compressa*	[219]
Burmannic acid (BURA)	Terpenoid acid	-Viability testing (MTT) -Flow cytometry (cell cycle, apoptosis) -Mitochondrial superoxide measurement, membrane potential -Western blot for caspases -DNA damage (γH2AX, 8-OH-dG -NAC used to demonstrate oxidative stress involvement	-BURA has selective anticancer potential, decreasing overall toxicity -Oxidative stress is becoming a targeted strategy in the development of new natural anticancer drugs -Requires preclinical/clinical studies to validate the effect	[220]
Silymarin	Flavonolignan	-In vitro assays on HSC-4, YD15, Ca9.22 lines (viability, Western blot, apoptosis) -In vivo studies (animal models) for tumor assessment and toxicity monitoring -Death receptor assays (DR5), caspase cascade	-Silymarin proves to be a promising oral anticancer agent, minimally toxic -Has the potential to be combined with other therapies, decreasing overall toxicity -Supports the use of natural phytotherapeutic compounds in oral oncology	[106]

Legend: LC3—microtubule-associated protein 1A/1B-light chain 3; p62 (SQSTM1)—sequestosome 1; PI3K/AKT—Phosphoinositide 3-kinase /Protein kinase B; NF-κB—Nuclear factor-κB; RAS/RAF/ERK—the most important signalling cascade among all MAPK signal transduction pathways, involved in cell proliferation and differentiation; E-FABP—Epidermal Fatty Acid Binding Protein; JNK 1/2—c-Jun N-terminal kinase 1 and 2; SREBP1—Sterol Regulatory Element-Binding Protein 1; α-SMA—Alpha-Smooth Muscle Actin; ZEB1—Zinc Finger E-box-Binding Homeobox 1; miR-200a—MicroRNA-200a; TWIST, SLUG—Transcription factors involved in EMT and cancer cell invasion; E-cadherin—a calcium-regulated adhesion molecule expressed in most normal epithelial tissues; EMT—Epithelial–Mesenchymal Transition; CDK2/4/6—Cyclin-Dependent Kinases 2/4/6; Mcl-1—Myeloid Cell Leukemia 1; GSH—Glutathione; PAI-1—Plasminogen Activator Inhibitor-1; c-Myc—a key regulator of cell proliferation, cell growth, differentiation, and apoptosis; IRE1α—Inositol-Requiring Enzyme 1 Alpha; Connexin 43—the most ubiquitously expressed member of the connexin family, facilitating intercellular communication; STITCH—Search Tool for Interacting Chemicals; Atg-5—Autophagy-related protein 5; Beclin 1—A key protein in the initiation of autophagy; 3-MA—3-Methyladenine; 8-OH-dG—8-Hydroxy-2′-deoxyguanosine; DR5—Death Receptor 5; RNA—Ribonucleic acid; qRT-PCR—Quantitative reverse transcription-polymerase chain reaction; YD10B, YD15—Human mucoepidermoid cancer cell lines; OSF—Oral submucous fibrosis; TNM—Classification of Malignant Tumours (Tumor, Node, Metastasis); CRP, IL-6—inflammation markers; NMR—Nuclear magnetic resonance; ESI-HRMS—Electrospray ionization-High resolution mass spectrometry; SAS, T.Tn—OSCC cell lines; HaCaT—Human adult keratinocyte cell line; AKT/mTOR—Protein Kinase B/mammalian target of the rapamycin; MAPK—Mitogen activated protein kinase; NAC—N-Acetylcysteine; PFT-α—Pifithrin-α; DEMU—Diethyl maleate and urethane; AP-1—Activator protein 1; SP-1—Specificity protein 1; FACS/P1—Fluorescence-Activated Cell Sorting/Subpopulation 1; AIF—Apoptosis inducing factor; ENDO-G—Endonuclease G; BALB/c mice- inbred strains in biomedical research; CDK—Cyclin-dependent kinase; TRET-OME—Treated oral mucosa equivalent; TIMP-2—Tissue inhibitor of metalloproteinases 2; KON—Human oral squamous cancer cell line; MRC-5—Human lung fibroblast cell line; GFP-LC3—Green fluorescent protein-Light chain 3; LDH—Lactate dehydrogenase; GNM—Genistein nanoformulation; TSCCa—Tongue squamous cell carcinoma‚ MP—Methylprednisolone; CTSS—Cathepsin S.

**Table 3:** Overview of phytochemical compounds formulation/combination and their mechanisms in oral cancer treatment.

Phytochemical Compounds Formulation/Combination	Class of Compounds	Methodology/Cancer Types	Mechanism of Action	The Importance of Results	References
Silymarin in nanostructured lipid carrier (NLC)	Flavonoid complex	-Statistics Box-Behnken design 33 for optimization NLC -Characterization of PS, PDI, %EE -In-situ gel tested for SME release -Test on KB cells: IC50, apoptosis (Sub-G0) -Comparison of free SME vs. SME- NLCs vs. in-situ gel	-Generates ROS, favoring apoptosis -Inhibits KB cancer cells (low IC50 value) -Increased penetration and sustained release to the mucosa	-SME-NLCs-Plx/CP-ISG mucoadhesive system offers an effective strategy for localized treatment of oral cancer -Improves bioavailability and reduces the required dose -Alternative/addition to chemo/radiotherapy	[109]
Polydatin (nanoencapsulation)	Stilbene derivative	-Syrian hamsters, DMBA induction for oral carcinogenesis -POL-PLGA-NPs administration -Tumor incidence, tumor volume measurement -Biochemical analysis (TBARS, LOOH, antioxidant enzymes) -Histopathology oral cavity -ANOVA, DMRT test	-Increases antioxidant enzymes (CAT, GPx, SOD) and non-enzymatic antioxidants (Vit. C, E, GSH) -Decreases Phase I enzymes (Cit P450, Cit b5) and increases Phase II (GST, GGT, GR) -Reduces lipid peroxidation and oxidative damage -Supports Nrf2, AMPK, and cell homeostasis	-Demonstrates the role of polydatin (nanoencapsulated) as an oral chemopreventive agent -Nanotechnology improves bioavailability and efficacy -Promising strategy for oral anticancer therapies with low toxicity	[221]
α-Mangostin (α-MG) (mucoadhesive film)	Xanthone	-α-MG mucoadhesive film formulation -MTT test on SCC25 (IC50~152.5 µg/mL) -HPV-16 pseudovirus test (attachment vs. post- attachment stage) -NO reduction test (RAW264.7 cells) -Fibroblast migration test (in vitro healing)	-Cytotoxic effect on SCC25 at conc. > 125 µg/mL -Inhibits the attachment of pseudovirus HPV-16 (not further steps) -Reduces NO production in macrophages (anti-inflammatory effect) -Promotes fibroblast migration, thus improving healing	-Potential agent for oral cancer, topical administration -May prevent HPV infection (oral cancer risk factor) -Anti-inflammatory and pro-healing effects -Possible clinical use in the management of oral cancer and associated lesions	[222]
Nanoformulated rosemary extract	Plant extract	-Obtaining chitosan NP with rosemary extract -Hep-2 lines (OSCC) -Cytotoxicity (MTT) -Cell cycle analysis, apoptosis -ROS measurement -Autophagy observation (TEM microscopy)	-Dose-dependent cytotoxic effect -Cycle arrest in G2/M phase -Increased ROS, cell death -Observation of autophagosomes (autophagy) -Chitosan encapsulation increases stability/bioactive extract	-Non-invasive method with potential to reduce the toxicity of standard therapy -Increases the efficacy of nanoparticle rosemary -Can be integrated in combination treatments for OSCC	[223]
Nanoemulsion-based orodispersible film of *Guava*	Plant extract formulation	-Nanoemulsion GLO:VCO, characterization (size, zeta potential) -Film formulation (1–30% nanoemulsion) alginate based -Testing of anticancer activity: IC50, colony formation, invasion, apoptosis (Annexin V) -Stability at 25 °C for 1 year	-Formation of GLO:VCO nanoemulsion (70:30) with droplets ~50 nm -Incorporation in orodispersible film (alginate) -Anticancer effect: inhibition of colony formation, migration, induction of apoptosis (Annexin V) -Stability 1 year, maintaining activity	-Non-invasive oral treatment, targeted directly at the tumor -Orodispersible film with nanoemulsion: easy administration while maintaining anticancer activity -Opens the way for new local anticancer pharmaceuticals with potential for clinical integration	[224]
Quercetin Formulation	Flavonoid	-Review of studies on quercetin in oral diseases -Discussion on nano/micro formulations, controlled systems -Integration of quercetin in topical products, gels, capsules, etc.	-Antioxidant, protection against oxidative stress -Antibacterial, anti-inflammatory (reduces gingival inflammation, etc.) -Oral anticancer (inhibits proliferation, induces apoptosis) -Innovative delivery systems (nanoparticles, controlled release) increase stability/absorption	-Great potential in the treatment of oral conditions, including OSCC -Modern formulations may improve stability and local penetration -Prospects for clinical and industrial research for dental/medical products	[44]
Thymoquinone (TQ) + Cisplatin	Quinone + Chemotherapy agent	-UMSCC-14C lines (oral cancer) vs. OEC (normal cells) -Cytotoxicity assays, short/long (6h+) -Expression assays p53, Bcl-2, caspase-9 -Apoptosis % compared to TQ, CDDP, combination	-CDDP: cytotoxic for both tumor and normal cells -TQ: selective cytotoxic, induces apoptosis via p53↑, Bcl-2↓, caspase-9↑ -TQ + CDDP combination: additive/synergistic effect, partially protects normal cells	-Thymoquinone can be an adjuvant for cisplatin, reducing the required dose -Increases treatment specificity, decreasing systemic toxicity -Opens prospects for the use of TQ in combination regimens in oral cancer	[225]
Anethole + Cisplatin	Phenylpropene + Chemotherapy agent	-Ca9-22 cells (oral cancer) -MTT, LDH, Hoechst assay (viability, cytotoxicity) -Wound healing, colony formation -Apoptosis assays (ΔΨm, caspases) -Western blot for MAPK, β-catenin, NF-κB	-Combination anethole + cisplatin: -Increase oxidative stress, apoptosis -Inhibits cell migration and growth, decreases colony formation -Blocks MAPK, β-catenin, NF-κB -Clear synergy, reducing cisplatin resistance	-Combination of anethole + cisplatin as a more effective and less toxic therapy -Future clinical applications supported by synergistic studies -Approach targeting multiple pro-tumor pathways	[226]
Apigenin + Oxaliplatin (OXA)	Flavonoid + Chemotherapy agent	-HSC-3 cells, groups: control, apigenin, OXA, combination -Wound healing, invasion assay, 3D culture (angiogenesis) -qPCR for LINC00857 -Proliferation, apoptosis analysis	-Low doses of OXA induce EMT (migration, invasion, angiogenesis) -Apigenin inhibits LINC00857 expression => blocks EMT -Combines the anticancer effect of apigenin with OXA, inhibiting proliferation + metastasis of OSCC cells	-Apigenin + OXA strategy prevents OSCC metastasis -LINC00857 becomes a molecular target -Possible safer treatment, reduced OXA doses, decreasing side effects	[227]
Quinic acid (QA)+Cisplatin	Hydroxycarboxylic acid + Chemotherapy agent	-MTT assay for cytotoxicity (QA and QA + cisplatin) -DAPI staining, RT-PCR (pro/anti-apoptotic gene expression) -Flow cytometry and Western blot for apoptosis validation, Akt signaling, cyclin D1	-QA reduces expression of anti-apoptotic genes -Inhibits cyclin D1 and Akt pathway result decreases proliferation -Increases the anticancer effect of cisplatin, favoring apoptosis	-QA can be an oral anticancer agent, potentiating cisplatin -Offers therapeutic alternatives and potentially lower doses of cisplatin, with low toxicity -Opens the way for preclinical/clinical studies	[228]
Resveratrol (RV) + Polydatin (PD)	Stilbene + Stilbene derivative	-In vitro studies on OSCC lines and in vivo (animal models) -Biodistribution, pharmaceutical (liposomes) evaluation -Synergy analysis with other chemotherapeutics -Observations on proliferation, metastasis, and apoptosis parameters	-RSV and PD demonstrate significant anticancer effect (in vitro/in vivo) -Liposomal formulations increase stability/efficacy -Synergistic effect with chemotherapeutics, reduces resistance -Low systemic toxicity	-Provides directions for the development of advanced delivery systems -Opens clinical perspectives for RSV/PD in OSCC -Potential to become a complementary therapy with minimal adverse effects	[229]

Legend: NLC—Nanostructured Lipid Carrier; SME—Silymarin Extract: ISG—In-Situ Gel; POL-PLGA-NPs—Polydatin-loaded Poly(lactic-co-glycolic acid) Nanoparticles; GLO:VCO—Guava Leaf Oil:Virgin Coconut Oil mixture; QA—Quinic Acid; OXA—Oxaliplatin; CDDP—Cisplatin; TQ—Thymoquinone; RV/RSV—Resveratrol; PD—Polydatin; PS—Particle Size; PDI—Polydispersity Index; %EE—Encapsulation Efficiency; DMBA—7,12-Dimethylbenz[a]anthracene; TBARS—Thiobarbituric Acid Reactive Substances; LOOH—Lipid Hydroperoxides; DMRT—Duncan’s Multiple Range Test; LINC00857—Long Intergenic Noncoding RNA 00857; HPV-16—Human Papillomavirus type 16; RAW264.7—Mouse macrophage cell line; NO—Nitric Oxide; Akt signaling—Intracellular pathway; Cyclin D1—Protein involved in cell cycle regulation; Nrf2—Nuclear factor erythroid 2–related factor 2; AMPK—AMP-activated Protein Kinase; EMT—Epithelial-Mesenchymal Transition; MAPK—Mitogen-Activated Protein Kinase; β-catenin—Protein involved in Wnt signaling; ΔΨm—Mitochondrial Membrane Potential; p53—Tumor suppressor protein involved in cell cycle arrest and apoptosis; Bcl-2/Bax/Caspase-9—Proteins regulating apoptosis; SubG0-; Plx/CP-ISG—Plexin inhibitor/Cisplatin-In situ gel; CAT—Catalase; GPx—Glutathione peroxidase; SOD—Superoxide dismutase; Cit P450—Cytochrome P450; Cit B5—Cytochrome B5; GST—Glutathione S—transferase; GGT—Gamma Glutamyl transferase; GR—Glutathione reductase; α-MG—alfa-Mangostin; TEM—Transmission electron microscopy; UMSCC-14C—Squamous cell carcinoma.

## Data Availability

No new data were created or analyzed in this study. Data sharing is not applicable to this article.
